# Silent Aggressor: Unveiling Large-Cell Duodenal Neuroendocrine Carcinoma

**DOI:** 10.7759/cureus.107486

**Published:** 2026-04-21

**Authors:** Shyamal Sheth, Shil Punatar, Tilemahos Spyratos

**Affiliations:** 1 Internal Medicine, Franciscan Health Olympia Fields, Olympia Fields, USA; 2 Gastroenterology, Franciscan Health Olympia Fields, Olympia Fields, USA

**Keywords:** duodenal cancer, duodenal ulcer disease, ki-67 expression, large cell neuroendocrine, spontaneous tumor lysis without precipitating factor

## Abstract

Duodenal neuroendocrine neoplasms (D-NENs) are uncommon tumors that account for a fraction of duodenal tumors. Although often incidentally discovered, those in the ampullary or peri-ampullary regions may present with symptoms due to biliary or gastrointestinal obstruction, including obstruction or bleeding. We describe a 65-year-old male who presented with fatigue, altered mentation, and melena. His evaluation was notable for severe anemia, metabolic derangements, and laboratory findings with concern for tumor lysis syndrome. Emergent management with hemodialysis and supportive care stabilized his condition. Esophagogastroduodenoscopy revealed a Forrest class 1B duodenal ulcer and histopathological examination of obtained biopsies confirmed a diagnosis of large-cell neuroendocrine carcinoma with a Ki-67 index of approximately 80%. Subsequent imaging demonstrated extensive hepatic involvement. Given the advanced stage of the disease and after multidisciplinary discussion, the patient elected against further oncologic intervention. This case highlights the diagnostic challenges and aggressive nature of high-grade D-NENs, emphasizing the importance of early recognition and a multidisciplinary treatment approach.

## Introduction

Duodenal neuroendocrine neoplasms (D-NENs) are rare tumors, comprising only 2-3% of primary duodenal neoplasms and less than 5% of all gastrointestinal neuroendocrine neoplasms [1]. Over recent decades, the incidence of these tumors has increased, with an estimated 1.1 cases diagnosed annually per 100,000 individuals in the United States [1]. D-NENs most commonly arise in the first or second portions of the duodenum and are frequently discovered incidentally during routine endoscopic evaluations. Tumors located in the ampullary or periampullary region, defined as arising within 2 cm of the ampulla of Vater, are more likely to be symptomatic and may follow a more aggressive clinical course.

Per the World Health Organization (WHO) 2022 classification, gastroenteropancreatic neuroendocrine neoplasms are divided into well-differentiated neuroendocrine tumors (NETs), graded G1-G3 based on the mitotic rate and Ki-67 proliferation index, and poorly differentiated neuroendocrine carcinomas (NECs), which are by definition high-grade. NECs, including large-cell and small-cell variants, carry a significantly worse prognosis and are characterized by markedly elevated Ki-67 indices, often exceeding 55% [2].

Clinically, D-NENs may present with abdominal pain, nausea, gastrointestinal bleeding, or obstructive jaundice, though many are discovered incidentally. The majority are non-functional, though rare functional tumors such as gastrinomas may produce hormonal syndromes. In high-grade NECs, spontaneous tumor lysis syndrome (TLS) represents a rare but life-threatening complication driven by rapid tumor cell turnover and has been reported in association with markedly elevated Ki-67 indices.

## Case presentation

A 65-year-old male presented with a one-month history of fatigue, altered mentation, and melena, preceded by a period of generalized malaise. On arrival, he was hypothermic and in acute distress. Physical examination was notable for jaundice, diffuse abdominal tenderness, and hepatomegaly.

Initial laboratory investigations revealed severe anemia (hemoglobin: 6.4 g/dL; reference range: 13.5-17.5 g/dL), hyperkalemia (8.4 mmol/L), hyperphosphatemia (14.8 mg/dL), elevated uric acid (22.9 mg/dL), and acute kidney injury with a serum creatinine of 18.9 mg/dL. Per the Cairo-Bishop criteria, the patient met three of four laboratory thresholds (hyperuricemia, hyperkalemia, hyperphosphatemia) with clinical evidence of renal dysfunction, fulfilling the diagnosis of clinical TLS (see Table 1). Notably, the absence of prior cytotoxic chemotherapy confirmed this as spontaneous TLS, a rare but life-threatening complication seen in high-grade solid tumors. The patient was admitted to the ICU and managed with hemodialysis, rasburicase, and blood transfusions.

**Table 1:** Laboratory Criteria for Tumor Lysis Syndrome (TLS) per the Cairo-Bishop Definition

Parameter	Cairo-Bishop Threshold	Patient’s Value	Meets Criterion?
Uric Acid	≥ 8.0 mg/dL	22.9 mg/dL	✔️
Potassium	≥ 6.0 mmol/L	8.4 mmol/L	✔️
Phosphorus	≥ 4.5 mg/dL	14.8 mg/dL	✔️
Calcium	≤ 7.0 mg/dL	8.5 mg/dL	❌
Creatinine Increase	≥ 1.5× baseline (AKI)	18.9 mg/dL (AKI)	✔️ Clinical TLS

**Figure 1:**
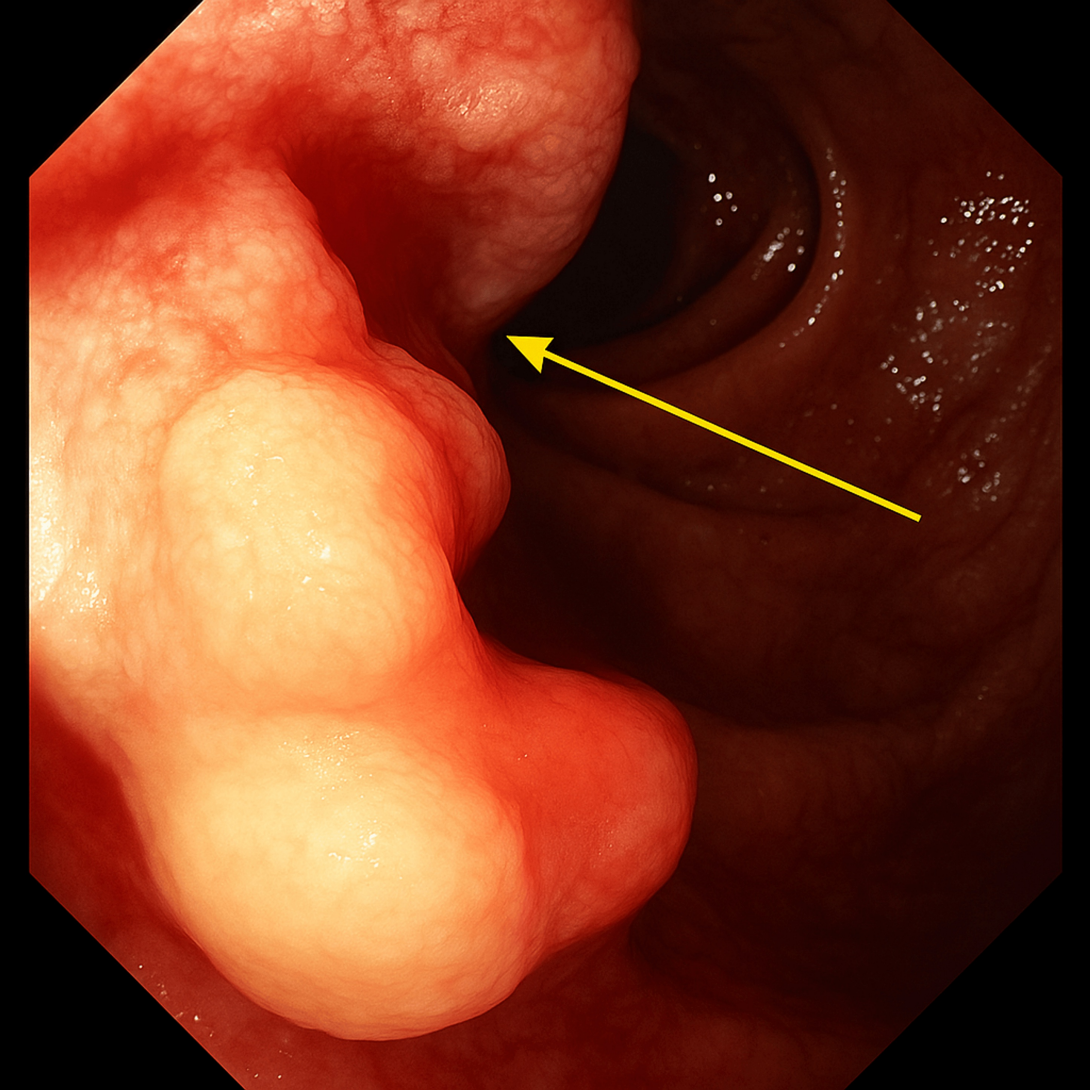
Anterior view of Forest Class 1B Ulcer

**Figure 2:**
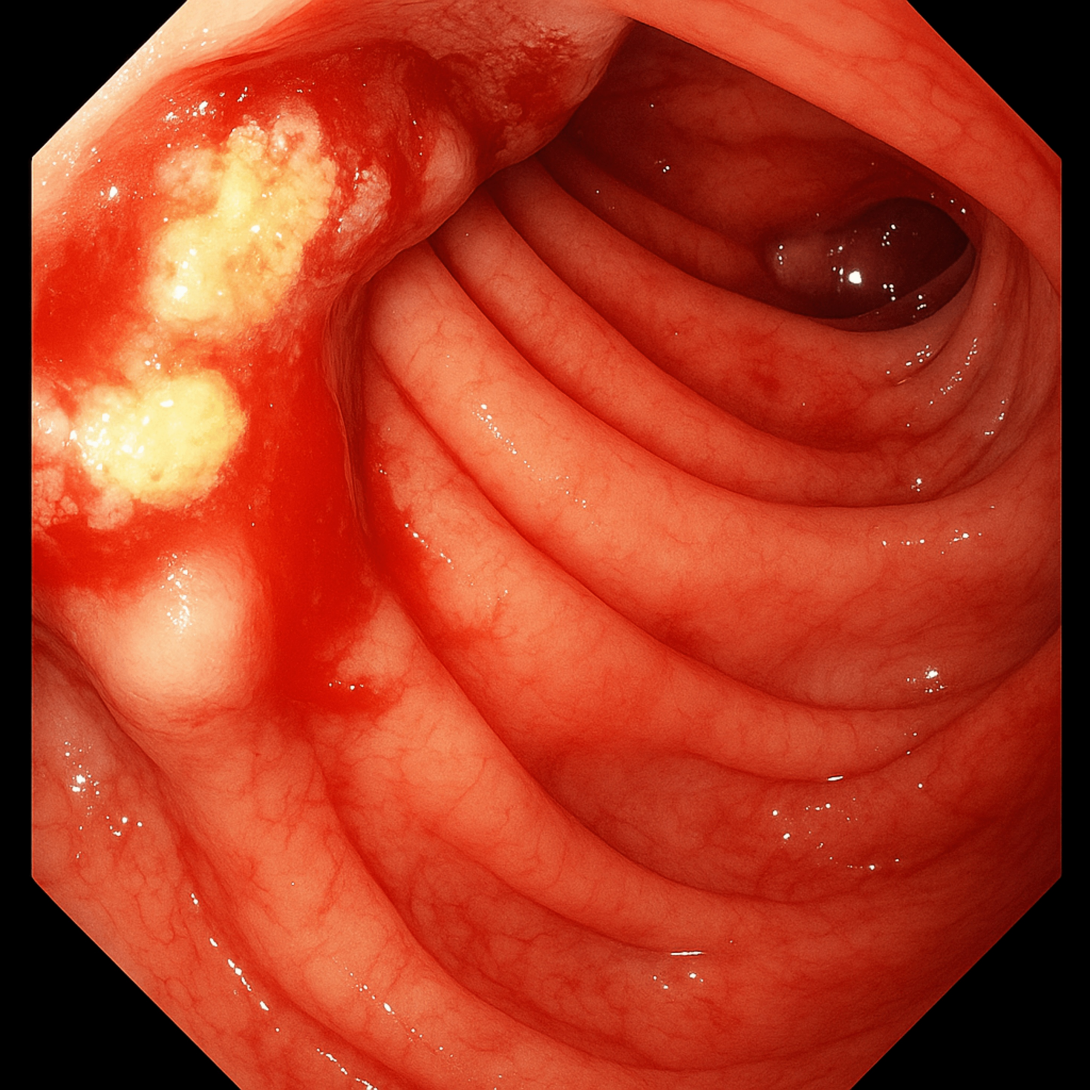
Retroflexed view of Forest Class 1B Ulcer

Following stabilization, esophagogastroduodenoscopy was performed, revealing a Forrest class 1B duodenal ulcer with active oozing hemorrhage (Figures 1, 2), which prompted tissue sampling. Computed tomography (CT) imaging demonstrated numerous hepatic lesions consistent with metastatic disease, as well as inguinal lymphadenopathy, reflecting the advanced stage at presentation. Histopathology confirmed large-cell neuroendocrine carcinoma (LC-NEC) with a Ki-67 proliferation index of approximately 80%. Following multidisciplinary discussion, the patient declined oncologic therapy.

## Discussion

This case illustrates the diagnostic challenges and therapeutic limitations associated with high-grade duodenal NECs. The patient's presenting symptoms of melena, anemia, and profound metabolic derangements reflect the aggressive biology of a poorly differentiated LC-NEC. A Ki-67 index of approximately 80% is consistent with the WHO 2022 classification of a high-grade NEC, characterized by rapid proliferation and significant metastatic potential [2-6].

While D-NENs are often asymptomatic and discovered incidentally, tumors involving the ampullary or periampullary region are more likely to present with overt clinical signs such as gastrointestinal bleeding or bile duct obstruction [2,5,7]. In this patient, melena and anemia prompted endoscopic evaluation, revealing a bleeding duodenal ulcer. CT imaging demonstrated numerous hepatic lesions consistent with metastatic disease, as well as inguinal lymphadenopathy, reflecting the advanced stage at presentation. The histological confirmation of LC-NECs underscores the critical role of tissue diagnosis in cases with ambiguous endoscopic findings.

TLS is most commonly observed following cytotoxic therapy in hematologic malignancies; however, spontaneous TLS, defined as TLS occurring in the absence of anticancer treatment, has been documented in high-grade solid tumors [8]. Its occurrence in NECs is exceptionally rare, with only a limited number of cases reported in the literature [8,9]. A review of published cases suggests that spontaneous TLS in NECs tends to occur in the setting of markedly elevated Ki-67 indices, bulky metastatic disease, and rapid tumor cell turnover, all of which were present in this patient [8,9]. Lactate dehydrogenase (LDH), a marker of cell lysis and tumor burden, is typically markedly elevated in TLS, with reported values in solid tumor-associated TLS often exceeding 1,000 U/L and in some cases surpassing 10,000 U/L [9]. While LDH values were not available in this case, the severity of the metabolic derangements, including a serum creatinine of 18.9 mg/dL, uric acid of 22.9 mg/dL, potassium of 8.4 mmol/L, and phosphate of 14.8 mg/dL, is consistent with the degree of tumor lysis reported in comparable cases. This patient fulfilled both laboratory and clinical TLS criteria per the Cairo-Bishop definition, and the absence of prior cytotoxic therapy confirmed the spontaneous nature of this complication [9]. Early recognition and rapid initiation of supportive therapy, including hemodialysis and rasburicase, are essential given the risk of fatal metabolic disturbances.

Management of D-NENs is guided by tumor grade, stage, and patient-specific factors. Surgical resection remains the preferred approach for localized, well-differentiated tumors. In contrast, LC-NECs typically present at advanced stages and carry a poor prognosis, with median overall survival reported at less than 12 months in metastatic disease [10]. Platinum-based chemotherapy, most commonly cisplatin or carboplatin combined with etoposide, represents the standard first-line systemic approach for metastatic NECs, though response rates remain modest and durable remissions are uncommon [11]. Emerging data on temozolomide-based regimens and immunotherapy combinations have shown some promise in selected patients, though evidence specific to duodenal primaries remains limited [12]. In this case, the patient declined systemic therapy following a comprehensive multidisciplinary discussion regarding prognosis and treatment burden, highlighting the importance of individualized, patient-centered decision-making in this setting.

## Conclusions

The LC-NEC of the duodenum is a rare but highly aggressive malignancy that frequently presents at an advanced stage, as illustrated by this case. The central novelty of this report lies in the occurrence of spontaneous TLS, a life-threatening metabolic emergency typically associated with hematologic malignancies, arising in the absence of any cytotoxic therapy. This underscores the extreme proliferative capacity of high-grade NECs and the need for clinicians to consider TLS in the differential when evaluating patients with high-grade solid tumors presenting with severe metabolic derangements. Early recognition, prompt histological diagnosis, and rapid initiation of supportive care are critical determinants of outcome in this setting. While systemic therapies may offer benefit in select patients, the overall prognosis remains poor, and individualized, compassionate care planning is essential. Given the single-case nature of this report, broader generalizability is limited, and further case series and prospective data are needed to better characterize the incidence, management, and outcomes of spontaneous TLS in NECs.
